# Motivation and Behaviour Change in *Parkrun* Participants in the Western Cape Province, South Africa

**DOI:** 10.3390/ijerph18158102

**Published:** 2021-07-30

**Authors:** Edgar Chivunze, Theresa L. Burgess, Fraser Carson, Kim Buchholtz

**Affiliations:** 1 Division of Physiotherapy, University of Cape Town, Cape Town 7935, South Africa; edgarphysio@yahoo.com (E.C.); theresa.burgess@uct.ac.za (T.L.B.); 2 Centre for Medical Ethics and Law, Faculty of Medicine and Health Sciences, Stellenbosch University, Cape Town 7505, South Africa; 3 Department of Sport and Exercise Science, LUNEX International University of Health, Exercise and Sports, 4671 Differdange, Luxembourg; 4 Department of Physiotherapy, LUNEX International University of Health, Exercise and Sports, 4671 Differdange, Luxembourg; kim.buchholtz@lunex-university.net

**Keywords:** physical activity, community, safe sport, social connectedness, well-being

## Abstract

Mass participation events are promoted in South Africa as a positive public health initiative. *Parkrun* has grown to be one of the most popular. The present study identifies the motives of residents in the Western Cape Province to join *parkrun* and how their involvement influences future physical activity levels. Participants (*N* = 1787) completed a survey consisting of demographic history, *parkrun* participation history, motivations for participation, and physical activity-related behaviour changes associated with *parkrun* participation. The majority of participants were female (*n* = 952) and over 50 years of age (median = 50; IQR = 38–59). Along with health-related benefits, the provision of a safe and organised event was reported as a key motive to participate. The social connectedness developed by *parkrun* encouraged continued participation and promoted uptake of more physical activity. Close to half the participants reported increases in physical activity levels after joining *parkrun*, which demonstrates the benefit obtained from participation in structured mass participation events. With the large diversity in socioeconomic status in South Africa related to physical activity levels, *parkrun* provides a protected and engaging environment that provides opportunity for increased physical activity and potentially reducing the burden on the healthcare system.

## 1. Introduction

South Africa is a low- to middle-income country (LMIC) with diverse cultures and people of different socioeconomic strata [1]. Physical activity levels are low compared to the global averages in other developing countries [2]. Joubert et al. [2] estimated a 49% physical inactivity rate in the adult population and that physical inactivity was the ninth highest cause of mortality in South Africa, with 3.3% of deaths directly attributable to inactivity. Further, non-communicable diseases (NCDs) can be attributed to 21.2% of deaths for women aged 30–70 and 32.3% of deaths for men aged 30–70 [3]. The health system has been overburdened in dealing with the increasing incidence of NCDs [4]. As the economic and social effects of these diseases together with physical inactivity increase, cost effective preventative measures to curb this trend are needed [5]. Physical activity is an affordable way to reduce the risks and manage NCDs [6]. Despite the focus of many public health initiatives aimed at improving participation in physical activity [7], many people are still not meeting the minimum thresholds of physical activity required for improving health outcomes. Globally, and in South Africa, efforts to reduce sedentary behaviour have included the development of mass participation physical activity events.

Mass participation physical activity events have been used to increase positive health behaviour among participants [8]. These are large-scale physical activity events organised for the public aiming to attract scores of people. Motivation for participation in these events could be for charity or to increase physical activity [9], with many events providing an inclusive and social environment [8]. One such event that introduced changes to the traditional way of conducting mass participation events is *parkrun*. *Parkrun* is a weekly free mass participation event held in parks and other free open areas [10]. In South Africa, since its inception in 2011, membership has grown to over 775,000 registered participants.

Research has identified participation in *parkrun* to be associated with positive improvements in both physical and mental health and well-being [11,12,13]. Stevinson and Hickson [12] noted that *parkrun* was important in engaging physically inactive participants; low socioeconomic communities were more likely to participate, and non-active runners used the events as an easy access starting point. Each event also creates a sense of belonging and social connectedness, which is a strong motivation for continued participation [14]. A sense of belonging is a common psychological need within self-determination theory (SDT) [15]. When applying SDT it can be determined that this increase in social connectedness will increase intrinsic motivation, which in turn will promote greater satisfaction and persistence [16]. Thus, a sense of belonging is potentially a key reason for the success of *parkrun* as a mass participation physical fitness event.

However, the majority of this research has been conducted in Australia and the UK [12,17], and overlooked the significant socio-cultural factors of other nations. The LMIC population, the wide variations in socioeconomic status, the diverse cultural practices, and high rates of crime in South Africa affect physical activity participation [18]. These are the factors that *parkrun* seeks to address as a mass participation event to encourage physical activity [19]. The aim of this study was, therefore, to identify the motives for participation in *parkrun*, and to ascertain the physical activity-related behavioural changes among registered *parkrun* participants in the Western Cape Province of South Africa.

## 2. Materials and Methods

### 2.1. Sample and Recruitment

Following formal permission from *parkrun* Global and *parkrun* South Africa, all registered members of the 37 *parkrun* South Africa sites in the Western Cape were contacted via email with details about the study, a plain language statement and a link to complete the online survey. Face-to-face collection was also conducted to include participants at three sites (Green Point, Kayamandi, and Zandvlei) who may not have had access to data or electronic resources required to complete the online questionnaire. Participants at these sites were recruited through short announcements made by *parkrun* organisers before the start of each event. After each race, interested participants completed informed consent and the survey in hard copy. Participants who completed the online questionnaire were not included in the face-to-face survey to prevent duplication of results and vice versa. To be eligible in either format, participants had to be over 18 years of age, registered with one of the regions of *Parkrun* sites, and to have completed a minimum of two *parkrun* events in the preceding six months.

### 2.2. Instruments and Measures

A survey, adapted from a previous *parkrun* study in Australia [17], was used to obtain demographic history, *parkrun* participation history, motivations for participation, and physical activity-related behaviour changes associated with *parkrun* participation. Minor adaptations were made to improve the contextual relevance of the questionnaire to South African *parkrun*, and included information on Discovery Health Vitality points. Discovery Health is South Africa’s biggest private medical aid covering healthcare costs. It rewards members who are registered in their Vitality Programme with points when they engage in health-/fitness-related activities, e.g., gym attendance, health screening, participating in *parkrun,* total daily step count, etc. For each *parkrun* event completed, members get 300 points. Once accrued, points can be redeemed for discounts on services and purchases. In an effort to improve response rates, the survey was compressed into the smallest functional size, and took on average four minutes to complete.

### 2.3. Procedure

Initially the survey was forwarded to experts in physical activity and wellness in public health for assessment of the content and construct validity related to the adaption of the original survey to a South African audience. Three experts gave feedback on the clarity and relevance of the questions and recommended the inclusion of socialisation as a motivation for participation and including comments for participants regarding motivation and physical activity before and after joining *parkrun*. The online version of the survey was completed over a two-week period in August 2019. Face-to-face data collection, using a paper-based survey that was identical to the online version, was completed at eight *parkrun* events over a five-week period in August and September of the same year. The study was conducted according to the guidelines of the Declaration of Helsinki, and approved by the University of Cape Town, Faculty of Health Sciences Human Research Ethics Committee (HREC REF 119/2019 and HREC REF 142/2019). A certificate of approval was also granted by the *parkrun* research board.

### 2.4. Data Treatment and Analysis

All face-to-face data collected were manually coded and saved for analysis. Informed consent was not provided by 627 participants and they were excluded, along with 169 who did not meet the inclusion criteria and 222 who had incomplete data. Statistical analyses were performed using TIBCO Software’s STATISTICA application version 13.5.0.17 and Graphpad Prism software version 8.3.0. The Shapiro–Wilk test was used to assess for normality among continuous variables of age, height, weight, and body mass index (BMI). All data were found to be not normally distributed. Categorical data were analysed based on frequency and grouped in relation to gender, educational level, level of physical activity, and motivation for participation. Associations between categorical variables were assessed using the Chi-square test of association. The Kruskal–Wallis H test was used to determine differences between at least three independent groups on total activity times (dependent variable). The Wilcoxon signed rank test and the Mann–Whitney U test were used to compare physical activity levels before and after joining *parkrun*. Independent variables used for this analysis include gender, education level, and gym membership. For all statistical analyses, a *p*-value less than 0.05 was accepted as statistically significant.

## 3. Results

A final sample consisted of complete data for 1787 participants. The majority of participants were female, aged 50 years or over, and had a BMI in the overweight category. Most participants were married, had completed higher education, were employed, reported to be in very good personal health, and were regular exercisers (see Table 1).

The provision of a safe environment was the greatest motivating factor for all participants, with the ability to accrue Discovery Health points second. Both physical and mental health factors motivated participants, with weight loss and stress relief significant contributing influences. Differences in motivation for participation in *parkrun* were compared, using Chi-square, according to sex. Women favoured a safe environment, stress relief, and cost as their main motivations, whereas men recognized competition with others as a key motivator (see Table 2).

Almost half (48%) took up new activities (*n* = 428) or increased their weekly physical activity levels (*n* = 429) after joining *parkrun.* All participants who reported increasing their physical activity levels after joining *parkrun* were grouped according to demographic characteristics (see Table 3). Most non-exercisers prior to commencing *parkrun* reported an increase in regular physical activity because of their participation.

Most participants reported health and fitness reasons to be important motivators for increasing physical activity post-*parkrun*, with weight loss and stress relief being key. Social connectedness and enjoyment were also prominent factors for continued participation (see Table 4).

## 4. Discussion

The aim of this study was to identify the motives for participant in *parkrun*, and to ascertain the physical activity-related behavioural changes among registered *parkrun* participants in the Western Cape Province of South Africa. Overall, *parkrun* has a positive impact on physical activity levels, which may reduce the burden on the health care system in the region. A greater number of participants were female (53.3%) and over the age of 50, which is comparable with *parkrun* participation globally [12,17]. Participants reported having a higher education qualification similar to previous studies on *parkrun* in Australia and the UK [13]. This is, however, higher than the national average of graduates in South Africa [20], which may imply that the economic activity of the Western Cape Province is greater, with a lower unemployment rate than other provinces of South Africa. Only 3% of participants in this study reported to be unemployed, significantly less than the 29% national unemployment rate (at the time of the study) [21]. Education level and employment status have been demonstrated to influence physical activity participation in LMICs [22] and may explain the motives and behaviours of the participants in this study.

Multiple motives exist for initiating participation in *parkrun*. There are considerable health and fitness benefits, including positive changes in weight, increases in cardiorespiratory fitness, and improvements in mental health [23,24]. Even small increases in physical activity have significant benefits on physical and mental health and well-being [25]. An additional benefit in South Africa is the potential to earn Discovery Health Vitality points, which in turn promotes increases in physical activity. Almost half of the participants in this study reported the ability to earn these points as a key motivation for participation. This can be used as a strategy to deal with barriers to exercise participation and to promote more physical activity in the region. These health and fitness benefits also increase motivation to continue participation in *parkrun* and initiate new physical activity practices, which is common within exercise motivation literature [26].

Many people chose *parkrun* as a safe environment for exercise participation. Over 79% of households in South Africa feel unsafe in their neighbourhoods [27]. *Parkrun* is a community event manned by community members and volunteers to ensure safety for participants. Women in particular identified this as a motive for participation. This is a consistent barrier to participation globally [28]. *Parkrun* addresses these issues and provides a safe space for exercise for all groups. It is not just a safer physical environment that is provided, but also a family-orientated nature [19]. The focus of *parkrun* is on fun, enjoyment, and personal development, which can break down recognised barriers to women’s participation in physical activity [26]. Further, there are no age limits or fitness requirements for entry [12].

The socialisation that is provided by *parkrun* is of great importance to continued participation. *Parkrun* creates a community and provides a sense of belonging. Over half the respondents in the present study reported socialisation to be influential to their participation. Similar findings have been attributed to *parkrun* globally [19]. *Parkrun* also promotes a system that encourages participants to volunteer in the organisation and running of each event. This approach supports more social interaction, with group identification and participation known to have a reciprocal relationship [14]. Consistent with SDT, the provision of social connectedness and a sense of belonging is important for the development of intrinsic motivation [15]. Applying the constructs of SDT further, *parkrun* participants are able to gain a sense of autonomy. Competence is also developed and supported by the recognition of achieving personal goals and reaching milestones of 50/100/150/etc. runs [19].

The large majority (83.4%) of participants were physically active (regular or occasional exercisers) prior to joining *parkrun*. Only a small percentage, much lower than the national average [20], classified themselves as non-exercisers. In South Africa, physical inactivity is as high as 49% for women and 43% for men [2]. The lower percentage in the current study may be attributed to the higher socioeconomic status of the region. Close to half of all respondents reported increases in physical activity levels since commencing with *parkrun*, with women most likely to incorporate more regular exercise and increased physical activity into their lifestyle. A similar finding has been reported in both Australia [13] and the UK [29]. Furthermore, of those who classified themselves as non-exercisers pre-*parkrun,* almost three quarters now integrated regular exercise into their daily life. This increased physical activity has the potential to reduce incidence of NCDs [30]. In LMICs the inclusion of mass participation events that promote socialisation in a safe, community environment, like *parkrun*, appears to be an important strategy to encourage less physically inactive lifestyles.

The Western Cape Province is one of the most economically active provinces of South Africa, contributing over 13% to the national gross domestic product and providing 23.6% of the country’s employment in 2018 [31]. As such, these results may not be generalisable to all the registered *parkrun* participants in the country due to the diversity of South African socioeconomic demographics. A logical first step would be to replicate this study in a greater proportion of the South African population to ascertain a more complete overview and minimize any selection bias. Furthermore, only active and registered *parkrun* participants were recruited and the results may not be applicable to non-*parkrunners* and those who had previously participated in *parkrun* and since withdrawn. The current study used a cross-sectional design and therefore, it is not possible to determine a cause-and-effect relationship in describing the various variables in the study. Although there were instances where people reported losing weight after *parkrun*, this could be as a result of other factors and not due to *parkrun* alone. A longitudinal prospective study is advised to evaluate health-related behaviour changes and motivations of *parkrun* participants.

## 5. Conclusions

In general, residents in the Western Cape Province of South Africa reported in this study that they participated in *parkrun* for physical and mental health benefits. The provision of a safe environment for physical activity by *parkrun* is an important factor, particularly in LMICs, and encourages greater participation from women and older people. The added dimension of social connectedness from an organised, regular mass participation event appears to increase self-determined behaviours and encourage persistence and continued physical activity. This may contribute to better health and reduce the load on the national health system. Almost half of all respondents reported increased physical activity since joining the *parkrun* community, demonstrating an opportunity for mass participation events to become an efficient method for improving the health and well-being of the general population.

## Figures and Tables

**Table 1:** Demographic characteristics of the sample.

Variable	Male	Female	All
*Sex (n)*	834	952	1787 ^+^
*Age (years)* ^#^	52 (40–62)	48 (37–58)	50 (38–59)
*Height (m)* ^#^	1.79 (1.74–1.83)	1.65 (1.60–1.70)	1.71 (1.65–1.78)
*Weight (kg)* ^#^	83 (75–92)	67 (60–78)	75 (65–86)
*BMI (kg*/*m^2^)*^#^	25.9 (23.7–28.7)	24.7 (22.1–28.1)	25.30 (22.9–28.4)
	***n (%)***	***n (%)***	***n (%)***
*Relationship status*			
Never married	172 (20.6)	222 (23.3)	394 (22.1)
Married	578 (69.3)	552 (58.0)	1130 (63.3)
Separated/Divorced	70 (8.9)	158 (16.6)	228 (12.7)
Missing	14 (1.7)	20 (2.1)	34 (1.9)
*Education level*			
No schooling	1 (0.1)	3 (0.3)	4 (0.2)
General education	7 (0.8)	5 (0.5)	12 (0.7)
Further education	122 (14.6)	136 (14.3)	258 (14.4)
Higher education	701 (84.1)	799 (84.0)	1500 (84.0)
Missing	3 (0.4)	9 (1.0)	12 (0.7)
*Employment status*			
Employed	588 (70.5)	679 (71.3)	1267 (71.0)
Working student	15(1.8)	27 (2.8)	42 (2.4)
Non-working student	31 (3.7)	25(2.6)	56 (3.1)
Retired	171 (20.5)	153 (16.1)	324 (18.1)
Unemployed	15(1.8)	40 (4.2)	55 (3.1)
Missing	14 (1.7)	28 (2.9)	42 (2.4)
*Gym member*	423 (50.7)	487 (51.2)	910 (51.0)
*Self-reported personal health status*			
Poor	3 (0.4)	5 (0.5)	7 (0.5)
Fair	44 (5.3)	49 (5.1)	93 (5.2)
Good	228 (27.3)	291 (30.6)	520 (29.1)
Very good	369 (44.4)	407 (42.8)	776(43.4)
Excellent	186 (22.3)	196 (20.6)	382 (21.4)
*Self-reported physical activity level*			
Non exerciser	125 (15.0)	154 (16.2)	279 (15.6)
Occasional exerciser	287 (34.4)	329 (34.6)	616 (34.5)
Regular exerciser	413 (49.5)	461 (48.4)	874 (48.9)

^+^ One person did not report sex. ^#^ Figures presented as median (interquartile range).

**Table 2:** Participant motivation to participate in *parkrun* according to gender.

Variable	Male *n (%)*	Female *n (%)*	χ²
Cost	278 (33.3)	444 (46.6)	32.68 **
Time	262 (31.4)	365 (38.3)	9.36 *
Weight loss	249 (28.9)	350 (36.9)	9.52 *
Stress relief	302 (36.2)	426 (44.7)	13.42 **
Safe environment	388 (46.5)	660 (69.3)	95.35 **
Competition with others	218 (26.1)	172 (18.1)	16.97 **
Earn Discovery Health points	366 (43.9)	463 (48.6)	4.03 *

* *p* < 0.05; ** *p* < 0.001.

**Table 3:** Post-*parkrun* physical activity increases categorised according to demographic characteristics.

Variable	*n* (%)	χ²	*p*
*Sex*			
Male	373 (44.7)	7.33	0.026 *
Female	485 (50.9)		
*Relationship status*			
Never married	214 (54.3)	13.65	0.009 *
Married	524 (46.4)		
Separated/Divorced	100 (44.0)		
*Education level*			
No schooling	3 (0.7)	4.16	0.77
General education	4 (3.3)		
Further education	124 (48.1)		
Higher education	719 (47.9)		
*Employment status*			
Employed	642 (50.7)	35.06	<0.001 **
Working student	25 (59.5)		
Non-working student	34 (60.7)		
Retired	116 (35.8)		
Unemployed	18 (32.7)		
*Self-reported personal health status*			
Poor	3 (37.5)	7.38	0.061
Fair	33 (35.5)		
Good	258 (49.7)		
Very good	382 (49.2)		
Excellent	179 (46.9)		
*Self-reported physical activity level*			
Non exerciser	203 (72.8)	135.3	<0.001 **
Occasional exerciser	343 (55.8)		
Regular exerciser	179 (46.9)		

* *p* < 0.05; ** *p* < 0.001.

**Table 4:** Association between motivation for *parkrun* participation and uptake of physical activity post-*parkrun*.

Variable	*n* (%)	χ²	*p*
Enjoyment (*n* = 1282)	643 (50.2)	9.37	0.002 *
Health/Fitness (*n* = 1538)	759(49.4)	7.30	0.007 *
Weight loss (*n* = 599)	328 (54.7)	18.13	<0.001 **
Stress relief (*n* = 728)	402 (55.2)	25.9	<0.001 **
Socialisation (*n* = 703)	372 (52.9)	11.78	0.001 *
Earn Discovery Health points (*n* = 829)	442 (53.2)	16.88	<0.001 **

* *p* < 0.05; ** *p* < 0.001.

## Data Availability

The raw data supporting the conclusions of this article can be made available on request by the authors. The data are not publicly available due to the level of consent provided by the participants on the use of confidential data.
